# Screening, brief intervention, and referral to treatment for drug- and alcohol-related health problems in emergency departments (EDs): review of outcomes, implementations, and costs

**DOI:** 10.1186/1940-0640-7-S1-A76

**Published:** 2012-10-09

**Authors:** Daniel Fischer, Dennis Donovan, Alyssa Forcehimes, Michael Bogenschutz

**Affiliations:** 1 Center on Alcoholism, Substance Abuse, and Addictions, University of New Mexico, Albuquerque, NM, USA; 2 Alcohol & Drug Abuse Institute, University of Washington, Seattle, WA, USA; 3 Department of Psychiatry, University of New Mexico CASAA, Albuquerque, NM, USA

## 

We conducted a literature review of screening, brief intervention, and referral to treatment (SBIRT) in emergency-department (ED) settings to evaluate intervention designs, outcomes, cost-savings, and implementation. Publications selected were primary or secondary research of SBIRT in ED settings for alcohol or drug use. Publications were separated into three categories: outcomes, costs, and implementation. Outcome literature excluded secondary research, focusing on primary analyses of SBIRT in ED settings that targeted drug and alcohol use. Twenty-six articles were found and grouped into interventions for alcohol or drugs. Some studies appeared in both groups. Of the 25 alcohol studies, 22 reported significant treatment effects. Of the 10 drug studies, eight reported significant treatment effects. Most studies reported outcome measures as change in alcohol or drug use; others identified treatment attendance or assessment scores as outcome variables. With regard to cost, nine publications outlined cost-savings, including reduced recurrent substance-related ED visits and reduced cost per patient. With regard to implementation, 22 publications discussed SBIRT implementation in EDs. Research suggests that physicians and mid-level providers can efficiently conduct SBIRT and address substance use disorders among ED patients. Although SBIRT has been successfully integrated into many busy EDs, it is a challenge requiring ongoing commitment, planning, and support from ED staff. Despite barriers to SBIRT implementation, EDs that have implemented SBIRT show cost savings and improved ability to diagnose and treat alcohol- and drug-related problems. Future research should explore the effect of SBIRT in EDs for drug-related problems as well as its long-term sustained implementation.

